# Evaluating TRAIL and IP-10 alterations in vaccinated pregnant women after COVID-19 diagnosis and their correlation with neutralizing antibodies

**DOI:** 10.3389/fimmu.2024.1415561

**Published:** 2024-09-03

**Authors:** Wei-Chun Chen, Shu-Yu Hu, Chao-Min Cheng, Ching-Fen Shen, Hui-Yu Chuang, Chin-Ru Ker, Der-Ji Sun, Ching-Ju Shen

**Affiliations:** ^1^ Institute of Biomedical Engineering, National Tsing Hua University, Hsinchu, Taiwan; ^2^ Division of Gynecologic Oncology, Department of Obstetrics and Gynecology, Chang Gung Memorial Hospital at Linkou, College of Medicine, Chang Gung University, Taoyuan, Taiwan; ^3^ Department of Obstetrics and Gynecology, New Taipei City Municipal Tucheng Hospital, New Taipei City, Taiwan; ^4^ International Intercollegiate Ph.D. Program, National Tsing Hua University, Hsinchu, Taiwan; ^5^ School of Traditional Chinese Medicine, Chang Gung University, Taoyuan, Taiwan; ^6^ Department of Pediatrics, National Cheng Kung University Hospital, College of Medicine, National Cheng Kung University, Tainan, Taiwan; ^7^ Department of Obstetrics and Gynecology, Kaohsiung Medical University Hospital, Kaohsiung Medical University, Kaohsiung, Taiwan; ^8^ Graduate Institute of Clinical Medicine, College of Medicine, Kaohsiung Medical University, Kaohsiung, Taiwan; ^9^ Department of Obstetrics and Gynecology, Pojen Hospital, Kaohsiung, Taiwan

**Keywords:** TNF-related apoptosis-inducing ligand, TRAIL, interferon gamma-induced protein 10, IP-10, COVID-19 vaccine, neutralizing antibody, Nab

## Abstract

**Background:**

This study evaluates tumor necrosis factor-related apoptosis-inducing ligand (TRAIL) and interferon-γ-induced protein-10 (IP-10) in pregnant women with COVID-19 and their newborns, exploring the effects of antiviral treatments and vaccine-induced neutralizing antibody (Nab) inhibition on these key viral infection biomarkers.

**Methods:**

We studied 61 pregnant women with past COVID-19 and either three (n=56) or four (n=5) doses of vaccination, and 46 without COVID-19 but vaccinated. We analyzed them and their newborns’ blood for TRAIL, IP-10, and Nab levels using enzyme-linked immunosorbent assays (ELISA), correlating these with other clinical factors.

**Results:**

Our study found lower TRAIL but higher IP-10 levels in maternal blood than neonatal cord blood, irrespective of past COVID-19 diagnosis. Cases diagnosed with COVID-19 < 4 weeks previously had higher maternal blood TRAIL levels (16.49 vs. 40.81 pg/mL, p=0.0064) and IP-10 (154.68 vs. 225.81 pg/mL, p=0.0170) than those never diagnosed. Antiviral medication lowered TRAIL and IP-10 in maternal blood without affecting Nab inhibition (TRAIL: 19.24 vs. 54.53 pg/mL, p=0.028; IP-10: 158.36 vs. 255.47 pg/mL, p=0.0089). TRAIL and IP-10 levels were similar with three or four vaccine doses, but four doses increased Nab inhibition (p=0.0363). Previously COVID-19 exposed pregnant women had higher Nab inhibition (p < 0.0001). No obvious correlation was found among TRAIL, IP-10, and Nab inhibition level.

**Conclusions:**

Our study suggests that lower maternal TRAIL and higher IP-10 levels compared to neonatal cord blood coupled with a rise in both markers following COVID-19 diagnosis that could be reduced by antivirals indicates a correlation to infection severity. Higher vaccine doses enhance Nab inhibition, irrespective of antiviral medication use and independent of TRAIL or IP-10 levels, highlighting the significance and safety of adequate vaccination and antiviral use post-diagnosis in pregnant women.

## 1 Introduction

COVID-19, caused by SARS-CoV-2, has rapidly spread worldwide, severely impacting global health and the economy (1). This disease particularly affects vulnerable groups, including pregnant women. Studies indicate that pregnant women with COVID-19 have a higher risk of antenatal or perinatal complications, including an increased rate of premature births (2). A systemic review revealed that 18% of pregnant women with COVID-19 experienced severe illness, exceeding the rates in the general population. The associated risks include preterm birth, preeclampsia, and emergent Cesarean delivery. Neonatal infections may also lead to increased fetal death and prolonged hospitalization (3). Vaccine development has been vital for protecting high-risk groups, including pregnant women, from severe COVID-19 (4). Our previous research shows that vaccinated mothers can transmit neutralizing antibodies (Nab) to their newborns, providing immunity against different subvariants of SARS-CoV-2, emphasizing the importance of maternal vaccination (5).

Research has identified interferon-γ-induced protein-10 (IP-10), tumor necrosis factor-related apoptosis-inducing ligand (TRAIL), and C-reactive protein (CRP) as key indicators for identifying viral infections and evaluating the severity of conditions such as COVID-19 (6). These markers effectively detect inflammation caused by viral infections (7). CRP, an acute-phase protein induced by IL-6 in the liver, is a sensitive biomarker for inflammation, infection, and tissue damage. Normally low in the bloodstream, CRP levels rise significantly within 12-24 hours after acute inflammation (8). TRAIL, a member of the TNF family, triggers cell apoptosis, including in immune and virus-infected cells, through its interaction with death receptors, serving dual roles in immune regulation (9). Conversely, IP-10, a chemokine produced in inflammatory responses, surges during infections. Triggered by interferon (IFN)-gamma, heightening IP-10 levels in response to viral infections prompts leukocytes and neutrophils to release substances that activate and draw B cells, T cells, and natural killer (NK) cells (10).

We previously published a study on the differences in TRAIL and IP-10 in pregnant women after receiving the COVID-19 vaccine (11). However, data on the differences in these values for pregnant women diagnosed with COVID-19 and their neonates are rare in the existing literature. Due to the absence of any similar research, we primarily aimed to investigate the levels of TRAIL and IP-10 in pregnant women diagnosed with COVID-19 by examining samples of both maternal and neonatal umbilical cord blood. Additionally, we also explored whether the use of antiviral medication at the time of diagnosis affected TRAIL and IP-10 levels. We also compared these findings with the levels of the same biomarkers in pregnant women who had not been diagnosed with COVID-19 in order to discern any potential differences in biomarker expression between these two groups. Additionally, we compared Nab inhibition rates among vaccinated pregnant women with and without COVID-19 infection diagnoses.

## 2 Materials and method

### 2.1 Participant collection and study design

**Figure 1:**
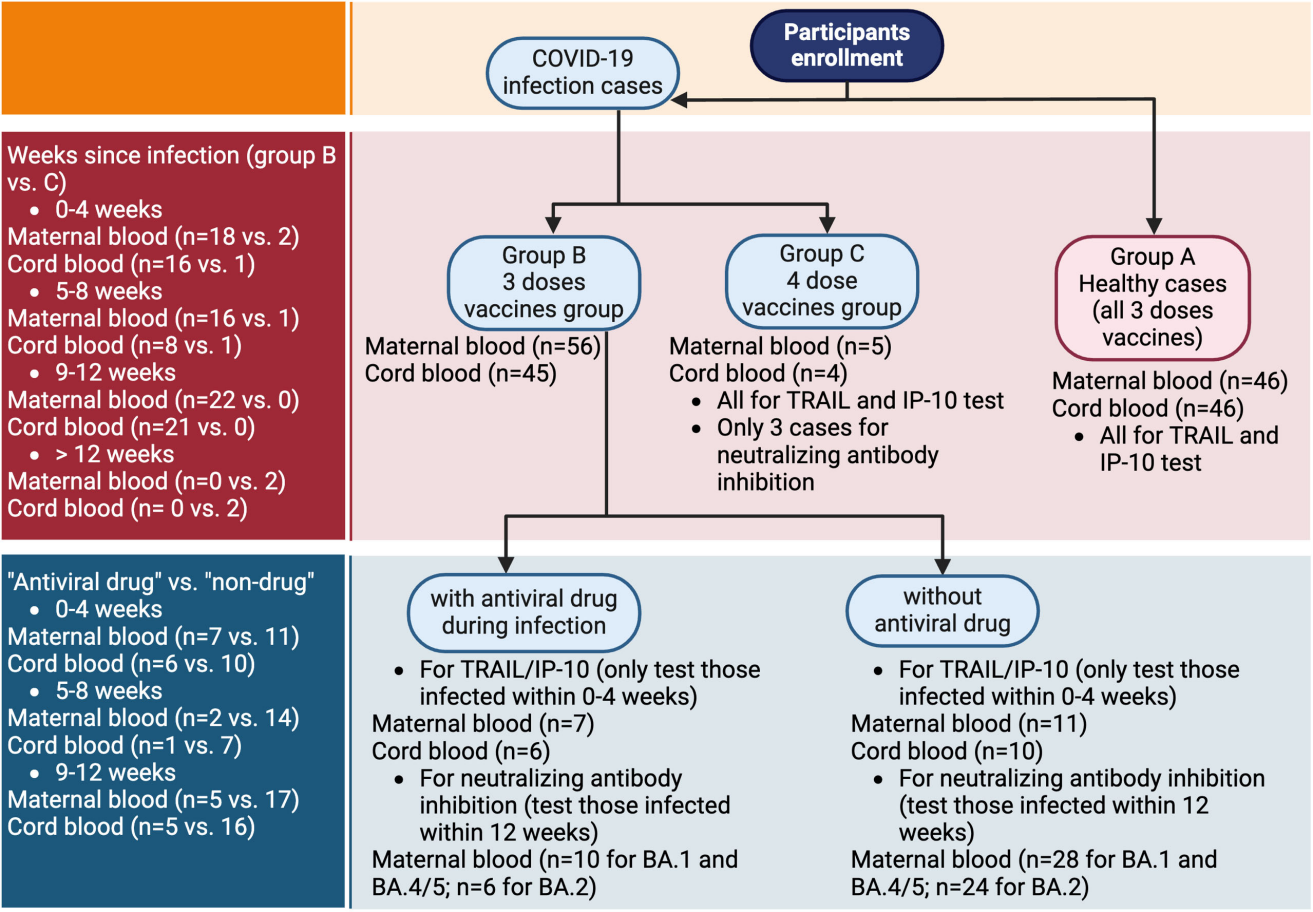
Flowchart of participants enrollment. TRAIL, TNF-related apoptosis-inducing ligand; IP-10, interferon gamma-induced protein 10.

The present investigation was conducted at the Kaohsiung Medical University Hospital, focusing solely on patients with singleton pregnancies. Our research involved a carefully selected group of participants, all of whom had been vaccinated against COVID-19, either prior to or during pregnancy. These participants were administered at least three doses of various COVID-19 vaccines, encompassing the Oxford/AstraZeneca ChAdOx1 nCoV-19 (AZD1222) vaccine, the mRNA-1273 Moderna vaccine, and the BNT162b2 vaccine. For those receiving vaccination during pregnancy, only mRNA-based vaccines, including Moderna and BNT162b2, were administered. Throughout the study, there were no significant reports of discomfort or health complications following vaccination. A particular focus of the study was that we also included participants who were previously diagnosed with COVID-19 during their pregnancy. Within this group, some were treated with the antiviral medication Paxlovid (Nirmatrelvir/Ritonavir), while others did not receive this treatment. Notably, among the pregnant women who contracted COVID-19, none exhibited severe complications. Compared to our previous work (11), the healthy group in this study, which has not been infected with COVID-19, partially overlaps with the participants in our previous study. However, in this study, they serve as the control group rather than the primary subjects. Additionally, while the previous study only analyzed maternal blood, this study includes analyses of both maternal blood and neonatal cord blood. The flowchart is depicted in **Figure 1**.

The study’s participants were all aged 20 years or older. We applied stringent exclusion criteria to ensure the integrity of our findings, eliminating any that had experienced or demonstrated one or more of the following: (1) preterm labor; (2) symptoms associated with COVID-19; (3) prior medical history indicating the need for immunosuppressant treatments; and, (4) pregnancy related morbidity including gestational diabetes or hypertension. This research was conducted after obtaining approvals from the local institutional review board (IRB), with the study being officially registered under IRB number KMUHIRB-SV(II)-20210087.

### 2.2 Sample collection

After obtaining informed consent from participants who met the inclusion criteria, peripheral blood from the mothers and post-clamping umbilical cord blood from the neonates were collected on the day of childbirth. These samples were promptly sent for advanced laboratory analysis. Additionally, relevant clinical data were extracted and documented from electronic medical records. Our investigation encompassed a range of maternal attributes, including age, body mass index (BMI), previous pregnancies, and gestational weeks. Neonatal factors, such as birth weight and gender, were also considered. Clinical vaccination data were gathered, encompassing the doses, dates, and types of COVID-19 vaccines administered. For participants with previous history of COVID-19 infection, we also verified the time of diagnosis and any antiviral medication used in their treatment. All of the aforementioned information was compiled for comprehensive analysis in subsequent investigations.

### 2.3 IP-10 and TRAIL concentration

In this study, our focus was on measuring two specific biomarkers, TRAIL and IP-10, in maternal blood and neonatal cord blood samples. The samples, initially stored at a temperature of -80°C, were later thawed to room temperature for analysis. We employed enzyme-linked immunosorbent assays (ELISAs) to determine the levels of TRAIL and IP-10, strictly adhering to the guidelines provided by the manufacturer (R&D Systems, Cat. No. DTRL00 and Cat. No. DIP100). ELISA plates were pre-coated with a monoclonal antibody specific to human TRAIL and IP-10. We subsequently added various concentrations of diluted serum and controls to each well. After a 2-hour incubation period, we washed away the unbound antibody-enzyme reagents, and incubated samples in the conjugate solution for 2 hours and then in the substrate solution for 30 minutes. Stop solution was added, and colorimetric change was quantitatively measured using an ELISA plate reader. We then used a specifically calculated saturation curve to convert those readings into actual sample biomarker concentration.

### 2.4 Neutralizing antibody inhibition test for SARS-CoV-2 omicron BA.1, BA.2, BA.5

We used an ELISA kit (Acro biosystems Cat. No. RAS-N056/RAS-N087/RAS-N107) to determine neutralizing antibody inhibition rate against SARS-CoV-2 Spike receptor binding domain (SRBD) in our samples. Human angiotensin-converting enzyme-2 (ACE2) protein was pre-coated on our ELISA plates, and both samples and controls were added followed by the addition of a solution containing horseradish peroxidase (HRP)-conjugated SRBD, specifically designed for various SARS-CoV-2 variants. Microplates were subsequently incubated for one hour at room temperature and shielded from light. After incubation for one hour, the supernatant was removed, we washed away unbound material, added substrate solution to each well and incubated for 20 minutes in the dark. To complete the process, we added stop solution, and quantitatively evaluated colorimetric values using an ELISA plate reader, reading absorbance levels at 450nm/630nm.

### 2.5 Statistics

In the present study, we employed analysis of variance (ANOVA) and chi-squared analyses to assess the levels and proportionate, characteristic variances among different cohorts. We also compared TRAIL, IP-10 levels, and Nab inhibition across various subgroups using ANOVA and the sample t-test. Additionally, we explored the interrelationships between TRAIL and IP-10 levels and their correlation to Nab inhibition for different Omicron subvariants of SARS-CoV-2 among our different subgroups via Pearson correlation methods. All statistical processing and analyses were carried out using SPSS Statistics (version 27, IBM, USA) and Microsoft Excel (Microsoft, Redmond, Washington, USA). We considered p-values below 0.05 as indicative of statistical significance. For the graphical representation of these statistical findings, we used GraphPad Prism software (GraphPad Software, San Diego, CA, USA).

## 3 Results

### 3.1 Participants characteristics

During the study period, we collected data from three distinct groups of pregnant women. Group A consisted of 46 healthy women who had never been diagnosed with COVID-19 and had received three doses of COVID-19 vaccine. Group B included 56 women who had been diagnosed with COVID-19 and also received three doses of the vaccine. Finally, Group C comprised 5 women who had been diagnosed with COVID-19 and had received four doses of the vaccine. The characteristics of these groups are detailed in **Table 1**. The mean age of participants was between 32 and 33 years, with a mean parity of approximately 1.3 to 1.4. The mean body mass index (BMI) was approximately 27 to 28, with Group C having a slightly higher mean BMI of 29.1. The mean gestational age at delivery for all groups was around 38 weeks. The interval between COVID-19 diagnosis and delivery was 6.68 and 11 weeks for Groups B and C, respectively. The interval between the last vaccine dose and delivery was 5.93, 30.79, and 19.0 weeks for Groups A, B, and C, respectively. The mean birth weights of the neonates were 3136.74 gm, 2978.0 gm, and 3092.5 gm for Groups A, B, and C respectively. In terms of neonatal gender distribution, in Groups A and B, 58% were male and 42% were female, while in Group C, 25% were male. Due to the limited availability of samples, only 45 neonatal cord blood samples from Group B and 4 from Group C were included in the subsequent analysis.

**Table 1:** Participants characteristics.

Variable		Three Doses (Group A)^a^	Three Doses (Group B)^b^	Four Doses (Group C)^c^
age of mothers		33.41*(± 5.19**)	32.79*(± 4.44**)	32.60*(± 8.09**)
Parity		1.39*(± 0.53**)	1.30*(± 0.56**)	1.40*(± 0.49**)
BMI		27.81*(± 4.29**)	27.10*(± 3.65**)	29.10*(± 4.30**)
weeks of gestation at delivery		38.60*(± 0.90**)	38.25*(± 0.87**)	38.60*(± 1.02**)
interval between the infection of COVID-19 and the collection of blood sample (day of delivery) (weeks)		–	6.68*(± 3.67**)	11.00*(± 11.37**)
interval between the first dose of COVID-19 vaccination and the collection of blood sample (day of delivery) (weeks)		37.69*(± 9.35**)	60.63*(± 11.31**)	77.80*(± 12.77**)
interval between the second dose of COVID-19 vaccination and the collection of blood sample (day of delivery) (weeks)		23.98*(± 7.32**)	49.54*(± 11.55**)	67.20*(± 10.46**)
interval between the third dose of COVID-19 vaccination and the collection of blood sample (day of delivery) (weeks)		5.93*(± 2.99**)	30.79*(± 13.41**)	48.80*(± 9.20**)
interval between the fourth dose of COVID-19 vaccination and the collection of blood sample (day of delivery) (weeks)		–	–	19.00*(± 13.13**)
Weight of newborn (g)		3136.74* (± 309.42**)	2978.00* (± 346.94**)	3092.50* (± 532.38**)
sex of newborn	Male Female	27(58%***) 19(42%***)	26(58%***) 19(42%***)	1(25%***) 3(75%***)

BMI, body mass index; *, mean; **, standard deviation (± SD); ***, percentage of all surveyed subjects; ^a^case number of Group A (3 doses-vaccinated cases without previous COVID-19 infection) as maternal blood=46, cord blood=46; ^b^case number of Group B (3 doses-vaccinated cases with previous COVID-19 infection) as maternal blood=56, cord blood=45; ^c^case number of four-dose group (all with previous COVID-19 infection) as maternal blood=5, cord blood=4.

### 3.2 TRAIL, IP-10, and neutralizing antibody inhibition levels at different intervals following COVID-19 diagnosis

**Figure 2:**
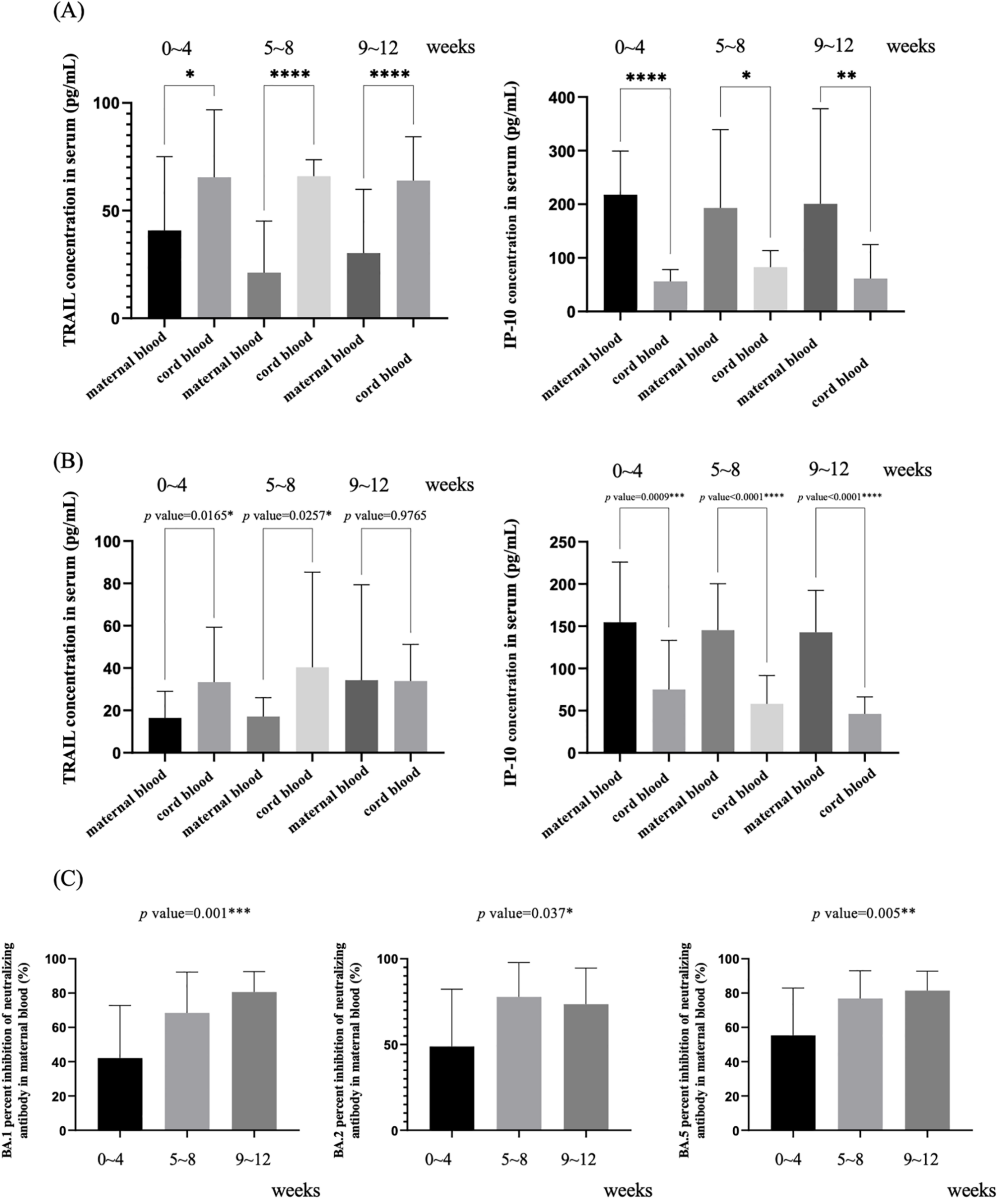
**(A)** Levels of TRAIL and IP-10 in maternal and umbilical cord blood at different intervals after previous diagnosis of COVID-19 in individuals who have received three doses of vaccine. **(B)** Levels of TRAIL and IP-10 in maternal and umbilical cord blood at different intervals after COVID-19 vaccination in those without previous COVID-19 diagnosis. **(C)** Neutralizing antibody inhibition rates at different intervals after previous COVID-19 diagnosis for BA.1, BA.2, and BA.5 in maternal blood from individuals who have received three doses of vaccine. TRAIL, TNF-related apoptosis-inducing ligand; IP-10, interferon gamma-induced protein 10; *p < 0.05; **p < 0.01; ***p < 0.001; ****p<0.0001.

In our study, we assessed the levels of TRAIL and IP-10, as well as Nab inhibition against Omicron subvariants BA.1, BA.2, and BA.4/5 SARS-CoV-2 in Group B, comprising individuals who had received three doses of COVID-19 vaccine and had a confirmed COVID-19 diagnosis. The data for these measurements at various intervals post-diagnosis are presented in **Supplementary Table S1**. **Figure 2A** illustrates the TRAIL and IP-10 levels from **Supplementary Table S1**. We observed that maternal blood consistently exhibited lower levels of TRAIL compared to neonatal cord blood, irrespective of the time elapsed since COVID-19 diagnosis. Conversely, levels of IP-10 were higher in maternal blood than in cord blood. However, the trends in TRAIL or IP-10 levels in either maternal or cord blood did not exhibit a fixed pattern over different time intervals, nor were there any significant differences observed.

To understand the impact of a COVID-19 diagnosis on TRAIL/IP-10 levels in maternal and cord blood, we also analyzed Group A, which consisted of participants who had received three doses of the COVID-19 vaccine but had not been diagnosed with COVID-19. The results are displayed in **Figure 2B**. When comparing the TRAIL/IP-10 levels at varying intervals since the last vaccine dose, we noted that within 8 weeks post-vaccination, maternal blood TRAIL levels were significantly lower than those in cord blood. However, beyond 9 weeks, there were no significant differences in these levels between maternal and cord blood. IP-10 levels in maternal blood were significantly higher than in cord blood, regardless of the time elapsed since the last vaccine dose.

**Figure 2C** presents the data on Nab inhibition from **Supplementary Table S1**. Regarding Nab inhibition against BA.1, BA.2, and BA. 4/5 Omicron subvariants of SARS-CoV-2, we observed that inhibition levels continued to increase over time post-diagnosis. The only exception was for BA.2, for which Nab inhibition levels between 9-12 weeks post-diagnosis were slightly lower than those between 5-8 weeks, although this difference was not statistically significant.

### 3.3 Antiviral drug impact on TRAIL and IP-10 levels in maternal and neonatal umbilical cord blood, and neutralizing antibody inhibition rates against BA.1, BA.2, BA.4/BA.5 in maternal blood

**Figure 3:**
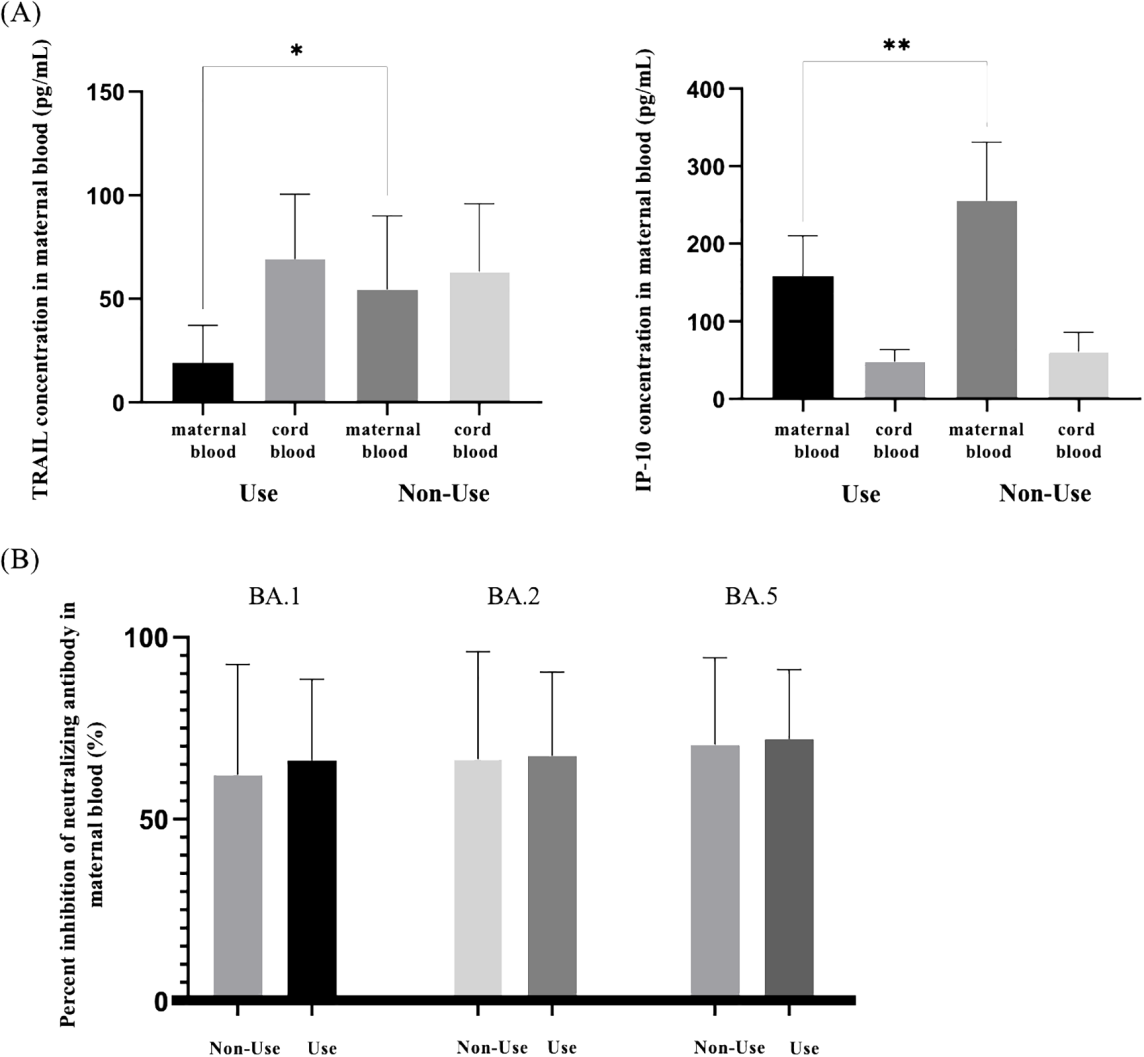
**(A)** Comparison of TRAIL and IP-10 in maternal and neonatal umbilical cord blood of individuals previously diagnosed with COVID-19 with and without the use of antiviral drugs. **(B)** Comparison of neutralizing antibody inhibition rates against BA.1, BA.2, and BA.5 in maternal blood among individuals previously diagnosed with COVID-19 with and without the use of antiviral drugs. TRAIL, TNF-related apoptosis-inducing ligand; IP-10, interferon gamma-induced protein 10; *p < 0.05; **p < 0.01.

Our study additionally investigated the impact of antiviral drug use on patients diagnosed with COVID-19 in relation to TRAIL and IP-10 levels, as well as Nab inhibition. Due to limited samples, we compared maternal blood and umbilical cord blood for TRAIL and IP-10 only in participants diagnosed with COVID-19 within four weeks. Additionally, for Omicron subvariants BA.1, BA.2, and BA.4/5 SARS-CoV-2, we compared maternal blood data within three months of diagnosis, with the results presented in **Supplementary Table S2**. **Figure 3A** illustrates the differences in TRAIL and IP-10 levels between patients diagnosed within one month with or without antiviral drug administration. **Figure 3B** displays the differences in Nab inhibition in patients diagnosed within three months, with or without antiviral drug administration.

Our findings indicated no significant difference in TRAIL and IP-10 levels in umbilical cord blood between those treated with antiviral drugs and those untreated (TRAIL: 69.24 vs. 63.21 pg/mL, p=0.7226; IP-10: 48.15 vs. 60.82 pg/mL, p=0.2844). However, in maternal blood, TRAIL and IP-10 levels were significantly lower in the cohort receiving medication compared to the untreated cohort (TRAIL: 19.24 vs. 54.53 pg/mL, p=0.028; IP-10: 158.36 vs. 255.47 pg/mL, p=0.0089). Regarding Nab inhibition, we observed no significant differences in response to Omicron subvariants BA.1, BA.2, and BA.4/5, irrespective of antiviral drug use in both cohorts.

### 3.4 TRAIL and IP-10 levels in maternal and umbilical cord blood, and neutralizing antibody inhibition rates against BA.1, BA.2, and BA.5 in maternal blood between individuals previously diagnosed with COVID-19 after receiving three and four doses of COVID-19 vaccine

**Figure 4:**
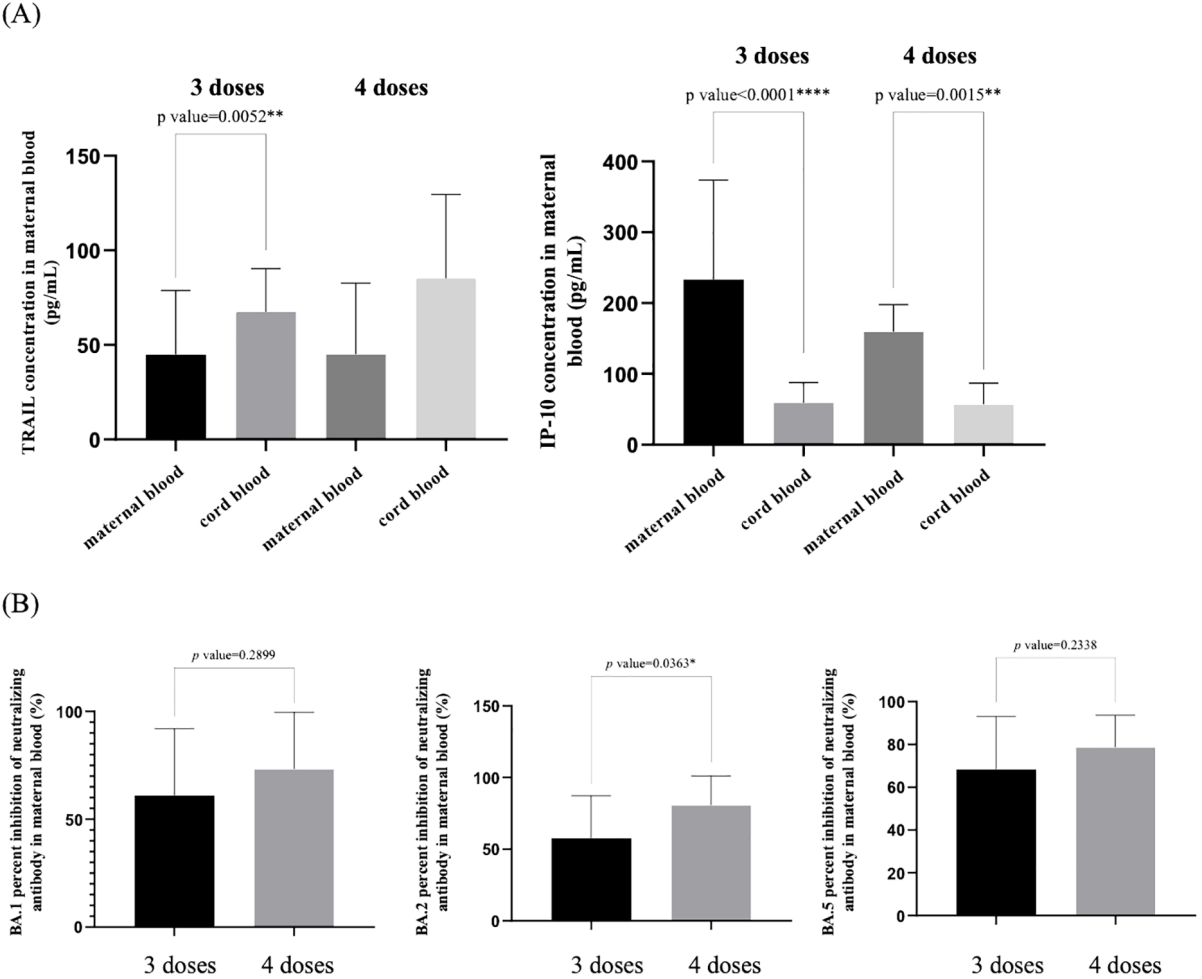
**(A)** Comparison of TRAIL and IP-10 levels in maternal and umbilical cord blood among individuals previously diagnosed with COVID-19 after receiving three and four doses of COVID-19 vaccine. **(B)** Comparison of neutralizing antibody inhibition rates against BA.1, BA.2, and BA.5 in maternal blood among individuals previously diagnosed with COVID-19 after receiving three and four doses of COVID-19 vaccine. TRAIL, TNF-related apoptosis-inducing ligand; IP-10, interferon gamma-induced protein 10; *p < 0.05; **p < 0.01; ****p < 0.0001.

We compared the levels of TRAIL and IP-10 in maternal blood and umbilical cord blood among pregnant women diagnosed with COVID-19, based on whether they received three or four doses of the vaccine, as shown in **Figure 4A**. Additionally, the difference in maternal blood Nab inhibition against Omicron subvariants BA.1, BA.2, and BA.4/5 following the administration of three or four vaccine doses is presented in **Figure 4B**. In this study, all subjects received at least one vaccine dose during pregnancy.

We observed that regardless of whether three or four vaccine doses were administered, maternal blood TRAIL levels were consistently lower than those in umbilical cord blood, but maternal blood IP-10 levels were higher than those in umbilical cord blood. However, there were no significant differences in TRAIL and IP-10 levels between the cohorts who received three or four vaccine doses, either in maternal or umbilical cord blood. Regarding Nab inhibition in maternal blood, the cohort that received four vaccine doses exhibited higher levels compared to those who received three doses. Although we observed no significant difference in Nab inhibition against Omicron subvariants BA.1 and BA.4/5 SARS-CoV-2 (BA.1: p=0.2899; BA.4/5: p=0.2338) among patients who received three or four vaccine doses, a significant difference was observed for BA.2 (p=0.0363).

### 3.5 TRAIL and IP-10 levels in maternal and cord blood, and neutralizing antibody inhibition rates in maternal blood: comparisons between A) individuals previously diagnosed with COVID-19 at different intervals post-diagnosis and B) individuals not previously diagnosed with COVID-19 at different intervals following final COVID-19 vaccination

**Figure 5:**
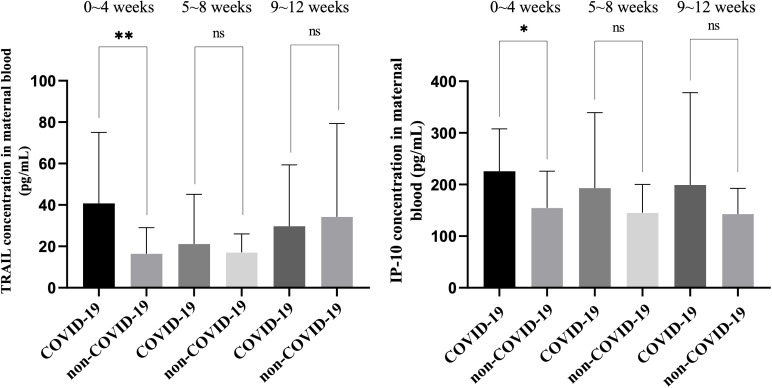
Comparison of TRAIL and IP-10 levels in maternal blood among individuals previously diagnosed with COVID-19 at different intervals after diagnosis and among individuals not previously diagnosed with COVID-19 at different intervals following final COVID-19 vaccination. TRAIL, TNF-related apoptosis-inducing ligand; IP-10, interferon gamma-induced protein 10; *p < 0.05; **p < 0.01; ns, non-significance.

**Figure 6:**
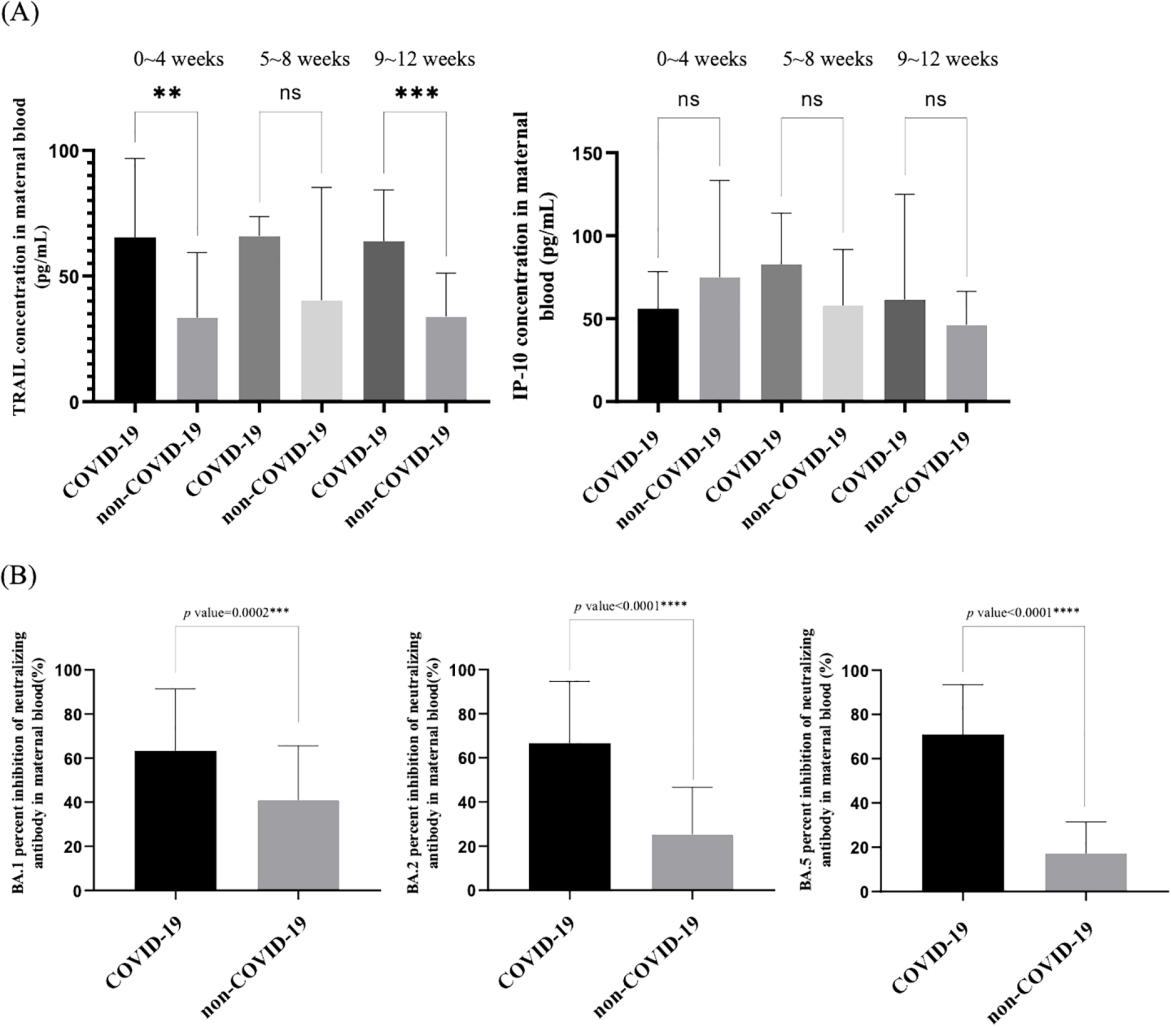
**(A)** Comparison of TRAIL and IP-10 levels in neonatal cord blood between individuals previously diagnosed with COVID-19 at different intervals after diagnosis and individuals not previously diagnosed with COVID-19 at different intervals following final COVID-19 vaccination. **(B)** Comparison of neutralizing antibody inhibition rates against BA.1, BA.2, and BA.5 in maternal blood between individuals previously diagnosed with COVID-19 and individuals not previously diagnosed with COVID-19. TRAIL, TNF-related apoptosis-inducing ligand; IP-10, interferon gamma-induced protein 10; **p < 0.01; ***p < 0.001; ****p < 0.0001; ns, non-significance.

We also compared the levels of TRAIL and IP-10 in maternal blood and umbilical cord blood at different time points post-COVID-19 diagnosis in pregnant women, with those who had not been diagnosed with COVID-19 and had received their last vaccine dose at various time points. The data for maternal blood are presented in **Supplementary Table S3** and **Figure 5**, while the data for umbilical cord blood are in **Supplementary Table S4** and **Figure 6A**.

Among the maternal blood comparisons, TRAIL levels within four weeks of COVID-19 diagnosis were significantly higher compared to those who were vaccinated but not diagnosed within the same timeframe (40.81 vs. 16.49 pg/mL, p=0.0064). Beyond five weeks, no significant difference was observed between the two cohorts (5 – 8 weeks: p=0.4775; 9 – 12 weeks: p=0.7113). Interestingly, maternal blood TRAIL levels decreased over time post-diagnosis, whereas in those not diagnosed but vaccinated, levels increased over time. Similarly, maternal blood IP-10 levels within four weeks post-diagnosis were higher compared to those who were only vaccinated within the same period (225.81 vs. 154.68 pg/mL, p=0.0170), with no significant difference observed beyond five weeks (5 – 8 weeks: p=0.1750; 9 – 12 weeks: p=0.2431). In contrast to TRAIL, maternal blood IP-10 levels decreased over time post-diagnosis, while in non-diagnosed but vaccinated individuals, the levels remained relatively stable over time.

Regarding umbilical cord blood comparisons, TRAIL levels were higher in diagnosed cases within four weeks and 9 – 12 weeks post-diagnosis compared to those who were vaccinated but not diagnosed within the same period (within 4 weeks: 65.47 vs. 33.34 pg/mL, p=0.003; 9 – 12 weeks: 63.94 vs. 33.90, p=0.0002). However, no significant difference was observed at 5 – 8 weeks. The TRAIL levels in umbilical cord blood did not change significantly over time, regardless of COVID-19 diagnosis. For IP-10, no notable difference was observed between diagnosed and non-diagnosed cohorts over time (0 – 4 weeks: p=0.2305; 5 – 8 weeks: p=0.0867; 9 – 12 weeks: p=0.4493), and the variation in IP-10 levels over time was not significant in either group (COVID-19 diagnosis: p=0.4231; non-COVID-19 diagnosis: p=0.2052).

Furthermore, for pregnant women who had received three doses of COVID-19 vaccine, the comparison of Nab inhibition against Omicron subvariants BA.1, BA.2, BA.4/5 between those diagnosed with COVID-19 and those not diagnosed is presented in **Figure 6B**. We observed that pregnant women with a history of COVID-19 demonstrated significantly higher Nab inhibition for all subvariants compared to those who had not been diagnosed with COVID-19.

### 3.6 The correlation between TRAIL, IP-10, and Nab inhibition rates in individuals previously diagnosed with COVID-19

We analyzed the correlation between maternal blood TRAIL and umbilical cord blood TRAIL, as well as between maternal blood IP-10 and umbilical cord blood IP-10 in pregnant women diagnosed with COVID-19, as shown in **Supplementary Figure S1**. We observed that the correlation values “|r|” were less than 0.1 without significant p values, indicating a low degree of correlation. Additionally, **Supplementary Figure S2** presents the correlation between maternal blood TRAIL and maternal blood IP-10, as well as between umbilical cord blood TRAIL and umbilical blood IP-10 in the same cohort. From this analysis, the correlation between maternal blood TRAIL and IP-10 was moderate with an “|r|” value of 0.4091 (p=0.0011), whereas in umbilical cord blood, TRAIL and IP-10 showed a low degree of correlation with an “|r|” value of 0.0001 (p=0.9992).

**Supplementary Figure S3** displays the correlation between maternal blood TRAIL and IP-10 and Nab inhibition against Omicron subvariants BA.1, BA.2, and BA.4/5. We found that the correlation with maternal blood TRAIL was significant for all three subvariants, but ranged from low to moderate (TRAIL to BA.1: r=-0.3206, p=0.0263; to BA.2: r=-0.4982, p=0.0011; to BA.4/5: r=-0.4358, p=0.0020). However, the correlation between maternal blood IP-10 and Nab inhibition was not statistically significant, with “|r|” values less than or equal to 0.1, indicating a low degree of correlation.

## 4 Discussion

Our research reveals that, regardless of a COVID-19 diagnosis, TRAIL levels in maternal blood are consistently lower than those in fetal umbilical cord blood, while IP-10 levels in maternal blood are consistently higher than in fetal umbilical cord blood. This observation has not been previously reported and the exact reasons remain unclear. TRAIL is also produced in human placenta and fetal membranes (12), and it is speculated to play a role in establishing immune tolerance at the feto-maternal interface during pregnancy (12). We hypothesize that this could be a result of an innate protective mechanism in the fetus, leading to higher levels of TRAIL to inhibit immune rejection, thereby maintaining a lower fetal immune-inflammatory response and lower IP-10 levels. However, these theories require further validation.

Furthermore, it has been noted that in pregnant women diagnosed with COVID-19, the levels of TRAIL and IP-10 in both maternal and umbilical cord blood are higher than in those not diagnosed with COVID-19. This effect is predominantly observed within one month after diagnosis of COVID-19; beyond one month, the levels of TRAIL and IP-10 in diagnosed and non-diagnosed individuals do not show significant differences. Post-treatment with antiviral drugs, the levels of TRAIL and IP-10 in maternal blood are lower compared to those who did not undergo antiviral treatment, whereas no significant difference is observed in umbilical cord blood levels regardless of medication. The effect of drugs on umbilical cord blood could be limited, possibly due to the placental transfer mechanism. However, because TRAIL and IP-10 are markers of COVID-19 infection, their levels might decrease under the influence of medication. Additionally, it has been observed that the use of medication does not significantly affect Nab inhibition.

Nab inhibition tends to increase with the administration of multiple vaccine doses, while TRAIL and IP-10 levels do not appear to be affected by the number of vaccine doses. Thus, TRAIL and IP-10 are more closely associated with infection indicators rather than vaccine dosage. Furthermore, among pregnant women who received the same number of vaccine doses, those who had been diagnosed with COVID-19 showed higher Nab inhibition, indicating that a prior diagnosis enhances the immune system’s memory effect. Overall, no significant correlation was observed between TRAIL, IP-10, and Nab inhibition, suggesting that these markers might exist independently of each other.

Tumor Necrosis Factor (TNF)-related apoptosis-inducing ligand (TRAIL), a member of the TNF family, can induce extrinsic induction of cell death when it binds with either of its two receptors that possess an intracellular death domain (13). TRAIL and its receptors are expressed in a variety of human tissues, especially in immune cells including lymphocytes, neutrophils, and macrophages (14). COVID-19 can increase the expression of TRAIL and its receptors in uninfected CD4+ and CD8+ lymphocytes, followed by a substantial reduction in lymphocyte count (15). TRAIL can induce apoptosis in macrophages and neutrophils, and therefore had anti-inflammatory and anti-atherosclerotic properties (16). The induction of apoptosis in normal dendritic cells (DCs), monocytes, and T cells by TRAIL is considered a form of immune regulation. Initially, TRAIL may play a role in immune suppression rather than eliminating viruses or virus-infected cells. However, in later stages, TRAIL might act to regulate the elimination of infected cells or limit viral replication in models of influenza or myocarditis virus infections, contributing to infection control (17). In COVID-19 infections, lower levels of TRAIL in the blood are associated with greater disease severity, including longer lengths of hospital and intensive care unit (ICU) stays (18).

TRAIL and its receptors can be detected in the amniotic membranes and amniotic fluid of pregnant women, and the concentration of TRAIL increases at delivery compared to samples from preterm births, regardless of labor status (19). TRAIL is known to be involved in the protection of vascular endothelial cells, and lower levels of blood TRAIL are associated with cardiovascular diseases and kidney disorders (20). In pregnant women, lower TRAIL levels may be related to early preeclampsia or hypertensive disorders of pregnancy (21). The comparison of TRAIL levels in maternal blood and umbilical cord blood of healthy pregnant women has not been specifically emphasized in past research. It is possible that higher TRAIL levels in fetal umbilical cord blood, compared to maternal blood, are a mechanism for the fetus to avoid maternal immune rejection, thereby modulating the intrauterine immune environment. However, the exact reasons and mechanisms require further study. This trend of higher fetal TRAIL levels compared to maternal TRAIL levels was not changed by any previous COVID-19 diagnosis. Additionally, TRAIL is indeed a protein associated with infection diagnosis. Thus, individuals on antiviral medication, who require less TRAIL to combat virus-induced inflammation, tend to have lower TRAIL levels than those not on medication. This observation has not been reported in past research but aligns with the previously understood functions of TRAIL.

IP-10 had been identified as indicative of COVID-19 disease severity. Elevated levels of IP-10 and monocyte chemoattractant protein-1 (MCP-1) in the serum of patients with severe COVID-19 have been noted, suggesting that these are biomarkers associated with the disease severity (22). Specifically, IP-10 levels were found to be significantly higher in COVID-19 patients compared to healthy individuals (667.5 vs 127 pg/mL, P <0.001). The increase in IP-10 may serve as an independent predictor of mortality in ICU patients (18). IP-10 is a chemokine rapidly and transiently induced following viral infections (23). The secretion of chemokine IP10 (also known as CXCL10) has been shown to trigger the activation and recruitment of monocytes, NK cells, and T cells to sites of tissue damage associated with infection (24). This chemotactic function is crucial for the protective immune response to infections. Studies have linked increased serum levels of IP10 and the secretion of recirculating cells upon re-stimulation with the severity of the disease (24).

Research also suggests a relationship between IP-10 and MCP-3 levels and the severity and progression of COVID-19 (22). Typically, IP-10 is transiently induced by Type I (α/β) or Type II (γ) interferons produced by DCs and T helper (TH) cells (25). Severe COVID-19 cases are also accompanied by T cell death, which is linked to plasma levels of soluble Fas Ligand (sFasL) and T cell apoptosis, with IP-10 being a characteristic feature related to disease severity (15). Furthermore, the gene for the IP-10 chemokine receptor, located in viral susceptibility regions such as CD26, can modulate activity (26). Thus, the increase in IP-10 levels during viral infections can be attributed to multiple mechanisms.

Regarding the association of IP-10 to pregnancy, studies have shown that the expression of IP-10 and its receptor CXCR3 is higher in the endometrial tissue of pregnant women compared to non-pregnant women. In mice prone to miscarriage, the levels of IP-10 in decidual tissues are lower than in those with normal pregnancies. This suggests that IP-10 is involved in the formation of a pro-inflammatory immune microenvironment during early pregnancy, regulating the distribution of immune cells and promoting the production of pro-inflammatory cytokines (27). Furthermore, patients with preeclampsia had significantly higher serum concentrations of CXCL10/IP-10 compared to women with normal pregnancies. Preeclampsia is characterized by an anti-angiogenic state and an enhanced systemic inflammatory response (28). This phenomenon can also be observed in pregnant women with acute pyelonephritis, where higher levels of IP-10 are found in maternal blood (29). Our study revealed that fetal umbilical cord blood exhibited lower levels of IP-10 compared to maternal blood, a finding that has not been previously reported. We speculate that this may be due to the uterine environment, where, during pregnancy, the fetus attempts to control the immune-inflammatory state to reduce immune rejection. However, the exact reasons and mechanisms for this require further investigation. Additionally, this trend remained unchanged regardless of any previous COVID-19 diagnosis.

Our data indicates that pregnant women diagnosed with COVID-19 exhibit higher levels of IP-10 compared to those who are not diagnosed, particularly within the first month of diagnosis. One month post-diagnosis, there were no significant differences in IP-10 levels, suggesting that the body’s inflammatory response decreases over time, leading to a natural reduction in IP-10 levels. Previous literature has also observed that IP-10 levels are elevated in pregnant women infected with SARS-CoV-2, and this elevation is correlated with the severity of the infection (30). Furthermore, the use of antiviral medication in diagnosed pregnant women resulted in a significant decrease in IP-10 levels, similar to the observed effects on TRAIL. The reduction in the virus-induced immune-inflammatory response due to medication correlates with a decrease in IP-10 levels. This finding has not been observed in previous studies. However, the same trend is not evident in the umbilical cord blood of newborns. The detailed reasons behind this discrepancy warrant further investigation.

TRAIL and IP-10 are commonly used as predictors of infectious disease severity, and in the context of COVID-19, the literature suggests that these biomarkers can serve as prognostic factors for COVID-19 severity (18). Although less emphasized in pregnant women with COVID-19, our study reveals that both TRAIL and IP-10 levels are elevated in patients within one month of COVID-19 diagnosis compared to those without a diagnosis, indicating a strong inflammatory response in the body. Furthermore, the use of antiviral medication results in a decrease in the levels of both biomarkers. Therefore, clinically, for pregnant women, it is advisable to use antiviral medication upon COVID-19 diagnosis to reduce maternal inflammation and minimize potential COVID-19 related complications. Consistent with previously published literature (5), pregnant women who have received more vaccine doses exhibit higher maternal Nab inhibition. Additionally, among those who received the same number of vaccine doses, those with a prior COVID-19 diagnosis showed even higher Nab inhibition. This value is not affected by the use of antiviral medication, and the trend of Nab inhibition is not influenced by TRAIL or IP-10 levels. Therefore, it is recommended that pregnant women receive sufficient vaccine doses, and in case of a COVID-19 diagnosis, antiviral medication should be used to mitigate potential complications.

There has been limited reporting in the past on the changes in TRAIL and IP-10 levels in pregnant women after COVID-19 infection. In addition to exploring this topic, this study is also the first to examine the changes in TRAIL and IP-10 levels following the receipt of antiviral medication to treat COVID-19 infection. Additionally, there has been a lack of literature addressing the comparison of TRAIL and IP-10 levels in maternal blood and neonatal umbilical cord blood in healthy pregnancies, which our study also reveals. The limitations of this study include an imbalance in the number of cases, with fewer pregnant women having received four vaccine doses. However, our data primarily focuses on those who received three doses, thus minimally impacting interpretation. Due to limited sample sizes, not all participants eligible for subgroup analysis could have their maternal or umbilical cord blood included, potentially introducing some bias. Furthermore, the vaccinated pregnant women in the study may have used the Oxford/AstraZeneca ChAdOx1 nCoV-19 (AZD1222) vaccine before pregnancy, and the Pfizer BioNTech (BNT162b2) COVID-19 vaccine or the Spikevax (elasomeran) COVID-19 vaccine (previously called the mRNA-1273 Moderna vaccine) during pregnancy. Although all vaccines were approved by WHO (Word health organization), the variety of vaccines regimens may lead to bias in the outcomes.

## 5 Conclusions

From our study, it is observed that among pregnant women, regardless of COVID-19 diagnosis, the levels of TRAIL are lower in maternal blood compared to fetal umbilical cord blood, while IP-10 levels are higher in maternal blood. In patients diagnosed with COVID-19 within a month, both TRAIL and IP-10 levels are higher than in those without a diagnosis, indicating a strong inflammatory response in the body. Additionally, the use of antiviral medication leads to a reduction in both of these biomarkers. Therefore, clinically, it is recommended that pregnant women diagnosed with COVID-19 use antiviral medication to reduce maternal inflammation and minimize potential COVID-19 related complications. Furthermore, pregnant women who received more vaccine doses exhibited higher maternal Nab inhibition. Among those receiving the same number of doses, those with a prior COVID-19 diagnosis have even higher Nab inhibition. This value is not affected by the use of antiviral medication, and the trend of Nab inhibition is not influenced by TRAIL or IP-10 levels. Therefore, it is advisable for pregnant women to receive sufficient vaccine doses, and in the case of a COVID-19 diagnosis, to use antiviral medication to alleviate potential complications.

## Data Availability

The original contributions presented in the study are included in the article/**Supplementary Material**. Further inquiries can be directed to the corresponding author.

## Supplementary material

Supplementary Figure S1 Levels of TRAIL and IP-10: the correlation between levels at maternal and umbilical cord blood from individuals previously diagnosed with COVID-19. TRAIL, TNF-related apoptosis-inducing ligand; IP-10, interferon gamma-induced protein 10.

Supplementary Figure S2 Correlation of TRAIL and IP-10 in maternal and umbilical cord blood in individuals previously diagnosed with COVID-19. TRAIL, TNF-related apoptosis-inducing ligand; IP-10, interferon gamma-induced protein 10.

Supplementary Figure S3 Correlation between levels of TRAIL/IP-10 and neutralizing antibody inhibition rates against BA.1, BA.2, and BA.5 in maternal blood from individuals previously diagnosed with COVID-19. TRAIL, TNF-related apoptosis-inducing ligand; IP-10, interferon gamma-induced protein 10; *, p < 0.05; **, p < 0.01.
