# Supplementary figures and images for: Evaluating TRAIL and IP-10 alterations in vaccinated pregnant women after COVID-19 diagnosis and their correlation with neutralizing antibodies
Source: Front Immunol. 2024 Sep 3;15:1415561. doi: 10.3389/fimmu.2024.1415561 (PMC11405216; doi:10.3389/fimmu.2024.1415561)

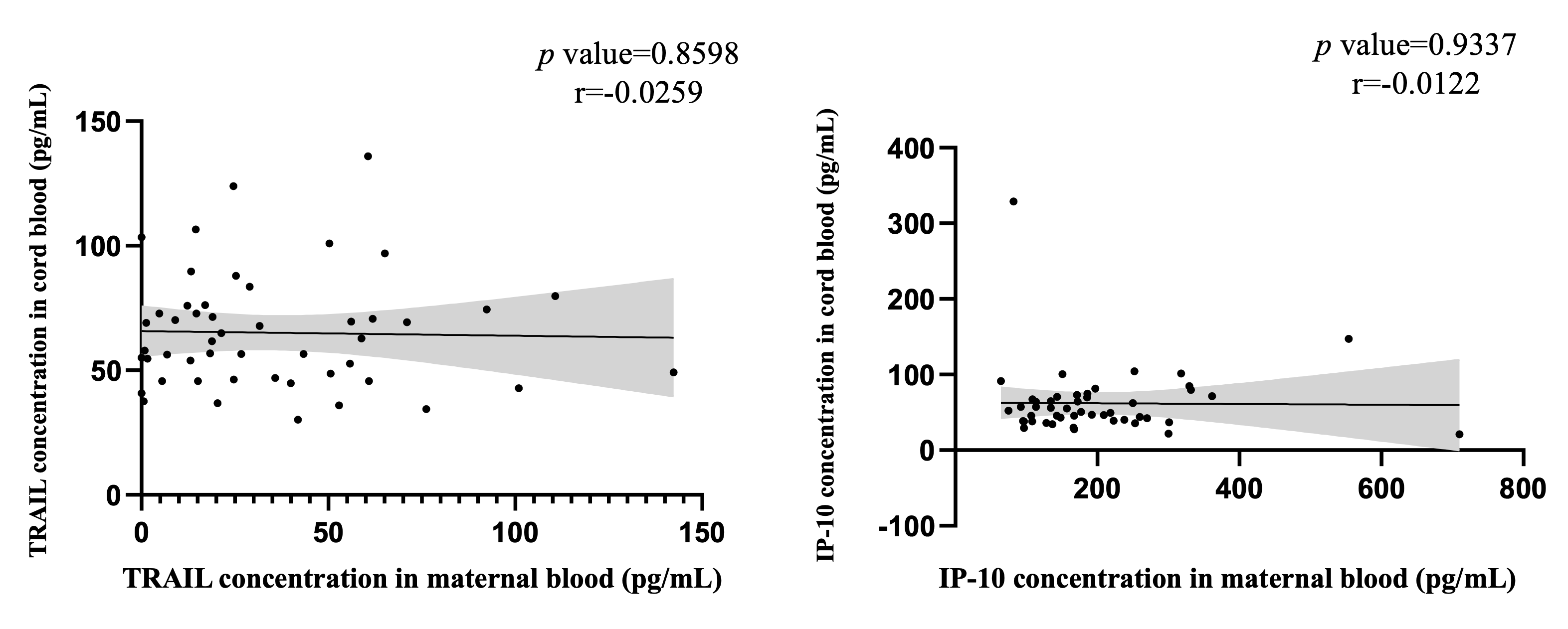

Supplement: Supplementary Figure S1 — Levels of TRAIL and IP-10: the correlation between levels at maternal and umbilical cord blood from individuals previously diagnosed with COVID-19. TRAIL, TNF-related apoptosis-inducing ligand; IP-10, interferon gamma-induced protein 10. [file Image1.tiff]

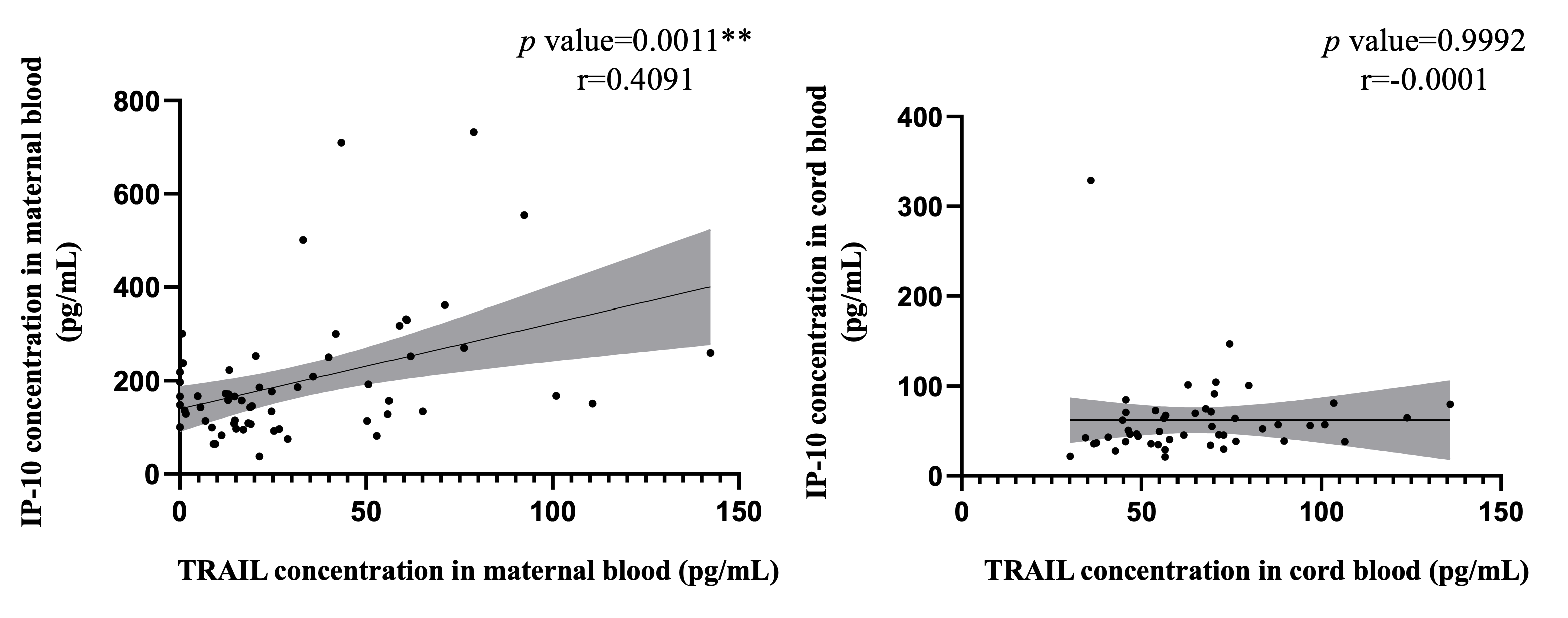

Supplement: Supplementary Figure S2 — Correlation of TRAIL and IP-10 in maternal and umbilical cord blood in individuals previously diagnosed with COVID-19. TRAIL, TNF-related apoptosis-inducing ligand; IP-10, interferon gamma-induced protein 10. [file Image2.tiff]

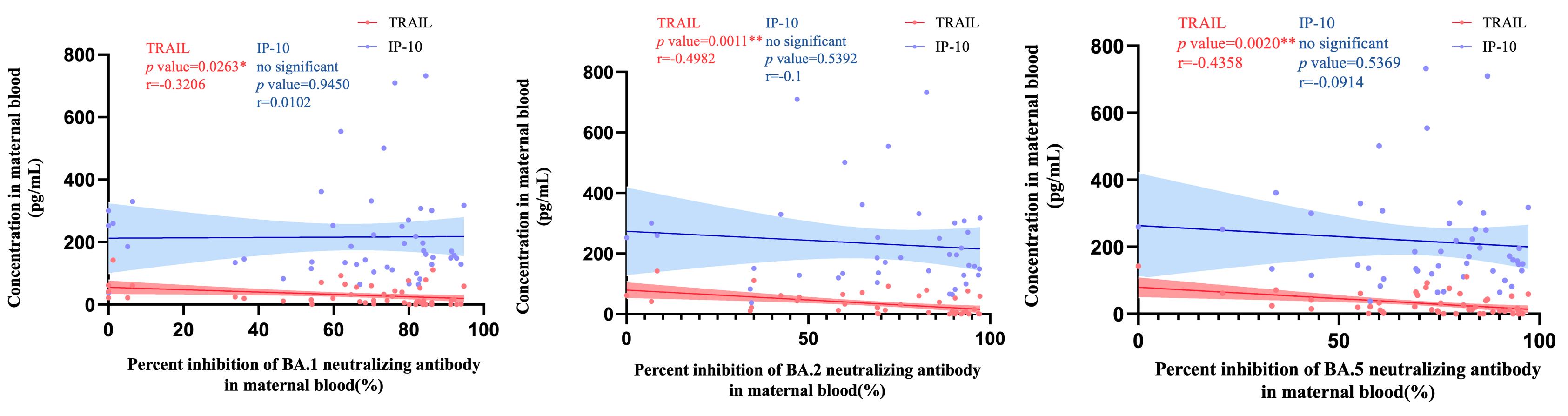

Supplement: Supplementary Figure S3 — Correlation between levels of TRAIL/IP-10 and neutralizing antibody inhibition rates against BA.1, BA.2, and BA.5 in maternal blood from individuals previously diagnosed with COVID-19. TRAIL, TNF-related apoptosis-inducing ligand; IP-10, interferon gamma-induced protein 10; *, p < 0.05; **, p < 0.01. [file Image3.tiff]
