# Supplementary material for: Evaluating TRAIL and IP-10 alterations in vaccinated pregnant women after COVID-19 diagnosis and their correlation with neutralizing antibodies
Source: Front Immunol. 2024 Sep 3;15:1415561. doi: 10.3389/fimmu.2024.1415561 (PMC11405216; doi:10.3389/fimmu.2024.1415561)
Supplement: Supplementary file 4 [file DataSheet1.docx]

**Table S1. TRAIL, IP-10, and neutralizing antibody inhibition levels at different intervals since COVID-19 diagnosis.**

| **Weeks Since Diagnosis，mean(SD)[n]*** | | | | | |
| --- | --- | --- | --- | --- | --- |
|  |  | **0-4 Weeks** | **5-8 Weeks** | **9-12 Weeks** | **p value** |
| **TRAIL (pg/mL)** | **maternal blood** | 40.81(33.27)[18] | 21.21(23.18)[16] | 30.27(28.91)[22] | 0.167 |
|  | **cord blood** | 65.47(33.27)[16] | 65.93(33.27)[8] | 63.94(33.27)[21] | 0.971 |
|  | **p value** | 0.0365 | <0.0001 | <0.0001 | - |
| **IP-10 (pg/mL)** | **maternal blood** | 217.71(79.31)[18] | 193.21(141.26)[16] | 200.69(173.38)[22] | 0.876 |
|  | **cord blood** | 56.07(33.27)[16] | 82.83(33.27)[8] | 61.40(33.27)[21] | 0.423 |
|  | **p value** | <0.0001 | 0.048 | 0.0015 | - |
| **BA.1 (%)** | **maternal blood** | 42.10(30.65)[13] | 68.46(23.86)[13] | 80.59(11.98)[12] | 0.001 |
| **BA.2 (%)** | **maternal blood** | 48.86(33.40)[10] | 77.82(20.09)[10] | 73.55(21.10)[10] | 0.037 |
| **BA.4/BA.5 (%)** | **maternal blood** | 55.40(27.54)[13] | 76.86(16.20)[13] | 81.46(11.32)[12] | 0.005 |

TRAIL, TNF-related apoptosis-inducing ligand; IP-10, Interferon gamma-induced protein 10; SD, standard deviation. * Only 45 cases among group B cases (3 doses vaccinated cases with previous COVID-19 diagnosis) had cord blood samples available. There were no 4 doses vaccinated cases (group C) here.

**Table S2. Comparison of TRAIL and IP-10 levels in maternal and neonatal umbilical cord blood, and neutralizing antibody inhibition rates against BA.1, BA.2, BA.4/BA.5 in maternal blood, between those who have and have not used antiviral drugs.**

| **Classification Based on Antiviral Medication Use，mean(SD)[n]** | | | | |
| --- | --- | --- | --- | --- |
|  |  | **Yes** | **No** | **p value** |
| **TRAIL (pg/mL)** | **maternal blood** | 19.24(17.99)[7] | 54.53(35.58)[11] | 0.028 |
|  | **cord blood** | 69.24(31.36)[6] | 63.21(32.73)[10] | 0.7226 |
|  | **p value** | 0.0042 | 0.5688 |  |
| **IP-10 (pg/mL)** | **maternal blood** | 158.36(51.86)[7] | 255.47(75.32)[11] | 0.0089 |
|  | **cord blood** | 48.15(15.42)[6] | 60.82(24.95)[10] | 0.2844 |
|  | **p value** | 0.0004 | <0.0001 |  |
| **BA.1 (%)** | **maternal blood** | 66.17(22.33)[10] | 66.24(30.34)[28] | 0.7113 |
| **BA.2 (%)** | **maternal blood** | 67.42(23.02)[6] | 66.58(29.43)[24] | 0.9488 |
| **BA.4/BA.5 (%)** | **maternal blood** | 72.08(19.03)[10] | 70.58(23.81)[28] | 0.8588 |

TRAIL, TNF-related apoptosis-inducing ligand; IP-10, Interferon gamma-induced protein 10; SD, standard deviation.

**Table S3. Comparison of TRAIL and IP-10 levels in maternal blood between previously COVID-19 diagnosed individuals at different intervals after diagnosis and previously non-COVID-19 diagnosed individuals at different intervals after final COVID-19 vaccination.**

| **Maternal Blood Levels of TRAIL and IP-10 in COVID-19 and non-COVID-19 pregnant Women，mean(SD)[n]*** | | | | | |
| --- | --- | --- | --- | --- | --- |
|  |  | **0~4 weeks**** | **5~8 weeks**** | **9~12 weeks**** | **p value** |
| **TRAIL (pg/mL)** | **COVID-19** | 40.81(34.24)[18] | 21.21(23.94)[16] | 29.77(29.59)[22] | 0.1654 |
|  | **non-COVID-19** | 16.49(12.58)[19] | 17.15(8.90)[21] | 34.33(45.01)[15] | 0.0800 |
|  | **p value** | 0.0064 | 0.4775 | 0.7113 |  |
| **IP-10 (pg/mL)** | **COVID-19** | 225.81(81.92)[18] | 193.21(145.89)[16] | 199.23(178.68)[22] | 0.8717 |
|  | **non-COVID-19** | 154.68(71.22)[19] | 145.37(54.87)[21] | 142.81(49.55)[15] | 0.8230 |
|  | **p value** | 0.0170 | 0.1750 | 0.2431 |  |

TRAIL, TNF-related apoptosis-inducing ligand; IP-10, Interferon gamma-induced protein 10; SD, standard deviation; *, For COVID-19 diagnosed cases, only 3 doses vaccinated cases were involved; **, Weeks for the COVID-19 diagnosed cases were from diagnosis to blood collecting. Weeks for the non-COVID-19 diagnosed cases were from last dose to blood collecting.

**Table S4. Comparison of TRAIL and IP-10 levels in neonatal cord blood between previously COVID-19 diagnosed individuals at different intervals after diagnosis and previously non-COVID-19 diagnosed individuals at different intervals after final COVID-19 vaccination.**

| **Cord Blood Levels of TRAIL and IP-10 in COVID-19 and non-COVID-19 pregnant Women，mean(SD)[n]*** | | | | | |
| --- | --- | --- | --- | --- | --- |
|  |  | **0~4 weeks**** | **5~8 weeks**** | **9~12 weeks**** | **p value** |
| **TRAIL (pg/mL)** | **COVID-19** | 65.47(31.30)[16] | 65.93(7.731)[8] | 63.94(20.35)[21] | 0.9709 |
|  | **non-COVID-19** | 33.34(25.95)[17] | 40.38(44.89)[18] | 33.90(17.27)[11] | 0.7962 |
|  | **p value** | 0.003 | 0.1267 | 0.0002 |  |
| **IP-10 (pg/mL)** | **COVID-19** | 56.07(22.20)[16] | 82.83(30.70)[8] | 61.40(63.56)[21] | 0.4231 |
|  | **non-COVID-19** | 75.04(58.14)[17] | 57.97(33.55)[18] | 46.23(20.07)[11] | 0.2052 |
|  | **p value** | 0.2305 | 0.0867 | 0.4493 |  |

TRAIL, TNF-related apoptosis-inducing ligand; IP-10, Interferon gamma-induced protein 10; SD, standard deviation; ***,** Only 45 cases among group B cases (3 doses vaccinated cases with previous COVID-19 diagnosis) had cord blood samples available; **, Weeks for the COVID-19 diagnosed cases were from diagnosis to blood collecting. Weeks for the non-COVID-19 diagnosed cases were from last dose to blood collecting.
